# Mapping Sustainable Tomato Supply Chain in Greece: A Framework for Research

**DOI:** 10.3390/foods9050539

**Published:** 2020-04-26

**Authors:** Foivos Anastasiadis, Ioanna Apostolidou, Anastasios Michailidis

**Affiliations:** Department of Agricultural Economics, School of Agriculture, Aristotle University of Thessaloniki, 541-24 Thessaloniki, Greece; ioanapost3@yahoo.gr (I.A.); tassosm@auth.gr (A.M.)

**Keywords:** food supply chain, agri-food sector, supply chain management, sustainability, value chain

## Abstract

Sustainable food supply chains are complex systems involving several stakeholders, processes, flow of goods/materials and information. The value generated in combination with the contradictory agendas among actors makes any groundwork for future research a challenging endeavor. Hence, an end-to-end mapping of the food supply chain under examination is a vital prerequisite for the design of a comprehensive research framework. This study exemplified such a mapping approach in the Greek sustainable tomato supply chain, providing significant insights for an impactful research agenda. Data were obtained from secondary sectoral sources and open interviews with key players across the supply chain—covering all its main stages, i.e., production, packaging, storing, transportation, wholesaling, and retailing. The findings are summarized in three supply chain maps that illustrate the areas concerning sustainability, value chain and stakeholders. These maps synthesize a bigger picture of the supply chain that reveals the complicated interactions among its actors, the hidden bottlenecks in the flow of information and the areas that need deeper exploration. Its fundamental implication is the design of a targeted research framework, underlying the main priorities of the Greek tomato supply chain and eventually the Greek agri-food sector.

## 1. Introduction

Tomato (*Solanum lycopersicum*) is among the most widely consumed vegetable crops globally. It is a nutritional food containing a substantial amount of vitamins, therefore, it plays an essential role in ensuring food security and nutrition [1]. Tomato is an essential ingredient in both the Greek and Mediterranean diet and it is consumed by the Greek population in large quantities, in many traditional dishes. Globally, among horticultural products, tomato ranks third for volumes of production—after potato and sweet potato—and first in terms of processing volumes [1,2]. Due to its high yields per hectare and its processing potential, it is one of the most important crops of the agri-food sector in Greece [3]. The total harvested tomato production in 2014 was 917,902 tons with a net production value of Int. $339,223,351.055. In 2014, according to FAOSTAT (Food and Agriculture Organization Corporate Statistical Database), Greece was among the top 20 countries of tomato production; while the first was China with 52,667,636 tons [4]. According to EUROSTAT, in 2018, Greece ranked seventh in Europe in terms of harvested tomato production with a volume of 885,150 tons; Turkey ranked the first with 12,150,000 tons [5].

Nowadays agriculture is facing severe challenges regarding resource efficiency, food production, and sustainability issues. Food security problems require a more efficient and sustainable production of nutritious food like tomatoes. In other words, innovation in the production systems must deliver more human value with the least environmental impact [6]. The need for sustainable agriculture is an increasingly crucial concept, which due to its complicated and multi-dimensional nature, should be considered from several angles [7]. In emergent agri-food supply chains, in which the adoption of sustainable practices is significantly low, the need for research and interventions is vital. A recent study in Greece investigated the sustainability performance, the penetration of technology, technological adoption and innovation in the agri-food sector, indicating very low levels of adoption, especially about sustainability and its social dimension [8]. The importance of social sustainability is also highlighted in Italy via the localized agri-food system (LAFS) and environmental social benefit strategies [9]. Another successful example is the 3S-model (Sustainable Smallholder Sourcing model) in which agribusiness multinationals can best include smallholders in their sourcing strategies and take social responsibility for large-scale sustainable and more equitable supply [10].

Remarkable changes and significant innovations have occurred in agriculture as the introduction of inert soil for the cultivation of greenhouse tomato production [11] and the rise of rooftop greenhouses (RTG) as an alternative to conventional rural agriculture [12], fostering sustainability, as a result. The technological innovation has also led to the establishment of climate control systems in greenhouses, improving productivity below the increment in cumulative energy demand, especially during the cold season, via heating systems in multi-tunnel greenhouses [13]. However, a significant number of studies tackling food security have adopted an energy–water–food (EWF) nexus perspective to assess food systems, as they are intrinsically linked to water and energy systems. They developed diverse methodologies that could guide decision-making to ensure sustainability and resilience in food availability via agent-based models [14] and using a life-cycle assessment (LCA) to evaluate the environmental performance [13,15,16].

The drying process in greenhouses seem to increase energy demand and reduce greenhouse gas emission with additional decline to the product losses, packaging materials, and transport volume, while it increases the product shelf life [17]. A research with reference to greenhouse gas emissions, showed that waste produced during the processing phase was the main contributor to the total environmental impact, followed by the packaging and cropping phase. It concluded that the mitigation options were composting and replacing mineral fertilization with compost, which greatly improved the environmental sustainability of the tomato supply chain [18]. Furthermore, there is a large potential in Europe for optimization in valorization of crop biomass in the food supply chain and useful actions such as reusing food waste. In the literature, there are strategies that could be employed for the processing and valorization of tomato side streams and waste fractions [19] and the results indicated that almost nothing was lost, because more than 85% of the total FLW (food loss and waste) were valorized into alternative sectors or activities [20].

Many firms have recognized the complexity of the supply chain due to the involvement of numerous stakeholders, yet its practical management remains very difficult. Particularly in the agri-food sector, supply chains have an awfully complex structure and for some products, it is much more problematic due to the nature of the relationships among stakeholders [21,22]. For instance, regarding the vital issue of information flows, even in simple dyadic buyer-supplier relations there is no full transparency [23]. Many key supply chain actors are not sharing information out of fear of being eliminated from the supply chain [24]. Similarly, bottlenecks in the flow of information in the agri-food supply chains result in minimal trust among stakeholders and eventually constrain further development of the sector [25]. Without any doubt, globally the supply chain complexity in every sector has increased continuously [26], and the supply chain visibility in either direction (upstream and downstream) is limited [27]. The same concept, if not in a greater extent, applies in the Greek agri-food sector. A 2019 study on the role of stakeholders in the sector, indicated, among others, that the situation in agri-food supply chains in Greece is rather complex [28].

Designing a targeted research framework, with a significant impact on making the supply chain (and eventually the agri-food sector) more sustainable and efficient must be among the top priorities of the agricultural research community. However, the complexity of the supply chain and the interactions among the stakeholders, as presented above, lessen the actual impact of former research designs/projects. End-to-end mapping of the supply chain is the vehicle to overcome this issue, since it unfolds the complicated structure of the supply chain and visually represents the interactions among its stakeholders. There are several definitions of a map from “a spatial representation of the environment…that stands for the environment that it portrays, and is both a likeness and a simplified model” [29] to “maps employ a form of visual language to communicate items of information” [30]. However, from a strict supply chain perspective, it is a fact that there is not yet a universal set of mapping conventions to represent a supply chain [31]. In practice, the essence of every supply chain map is strongly related to its goals and purpose. Among others, this purpose could be—material flow representation [32]; an integrated modeling framework for logistic chains [33]; development of a new framework on value creation [34]; mapping the value stream in the whole supply chain (SC) [35,36,37,38]; data flow diagram for SC [39]; product flow diagram [40]; and SC representation [41,42,43,44].

The objective of the current study is to employ a mapping approach in the Greek sustainable tomato supply chain. With the complicated nature of this scope in mind and the literature discussion above, the following research questions were formulated. What is the structure (product–material–value flows and stakeholders) and the key issues (interrelations and interactions) in the Greek sustainable tomato supply chain? In the following section, we present the methodological approach employed, data, and research design. In section three we present and discuss the results, three supply chain maps from a sustainability, value chain and stakeholders’ standpoint. The paper ends with the main mapping conclusions, the insights and recommendations.

## 2. Materials and Methods

### 2.1. Mapping Supply Chain Theoretical Foundations

From a methodological perspective initially, in 1997, the approach of value stream mapping by Hines and Rich was introduced [45] but since then it has been further developed by several researchers. The complicated nature of the supply chains and the everchanging environment, as discussed above in the literature review, has resulted in additional needs and has supported the evolution of mapping. Primarily, it referred material and information flow mapping mainly used to design production systems embracing the lean concept [46]. This approach was mainly used in the automotive industry, specifically by Toyota. Due to its simplicity, it was extensively applied to processes that require performance improvement [47]. As an extension of the mapping process, supply chain mapping provides a clear view and understanding of both, supply chain entities and the entire chain dynamics [48]. The supply chain consists of both a physical and support dimension, thus, the necessity for a deeper understanding and potential mapping of the supply chain [27]. Gardner and Cooper defined supply chain map as a visual representation of the linkages and entities of a supply chain, and all of the process and decision points that occur throughout a supply chain [31]. Additionally, the role of supply chain stakeholders was stressed on by expressing mapping as the process that illustrates the different entities that are connected by the material flow and the relationships between entities [49]. Thus, a supply chain map can contribute significantly on the planning process, can be a fundamental tool for implementing strategy, can also provide a supply chain interrelationships framework, but does not offer all the necessary details that allow the management of the supply chain [50].

Nonetheless, such techniques have not been applied for a long time in the agri-food sector, which has several distinct, and possibly unique, characteristics and circumstances, for example, the complicated role of stakeholders in the Greek sustainable agri-food supply chains [28] or the flow of information constraints in organic food emerging supply chains [25]. In response to this, Taylor [38] proposed a value chain analysis staged approach, in which each element was designed to boost and facilitate the implementation of procedures that would achieve quantifiable operational improvement. These seven stages were—(i) creating an understanding of the business potential of value chain analysis; (ii) understanding the supply chain structure and selecting a target value stream; (iii) analysis of the individual facilities along the chain; (iv) developing the current state map of the whole value chain; (v) analysis of issues, and opportunities along the whole chain; (vi) development of the whole chain future state map and recommendations; and (vii) creating a receptive organizational context.

### 2.2. Data and Research Design

**Figure 1:**
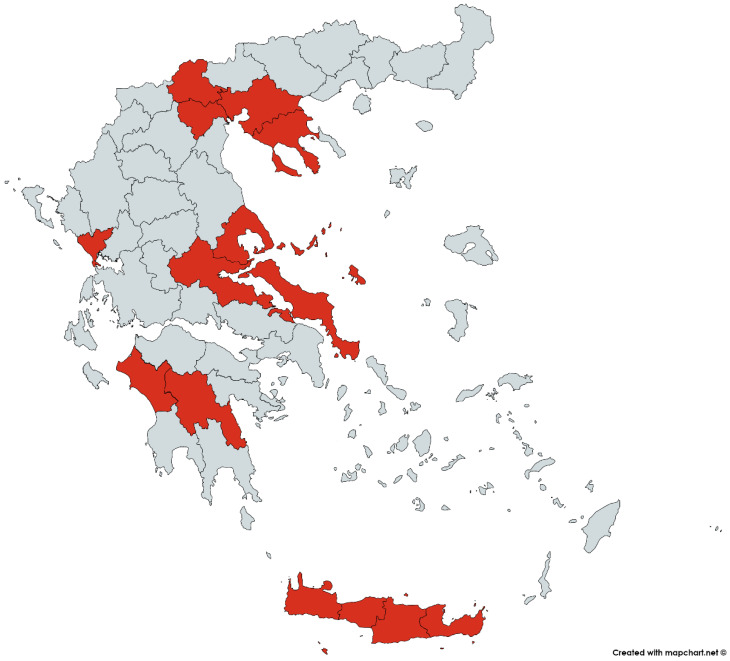
Regions with intense tomato production (Source: based on data from Hellenic Statistical Authority (ELSTAT) [56]).

In the current study, the sustainable tomato supply chain mapping involved secondary sectoral data [2,3,4,5,51,52,53,54] and subject matter expert interviews; for example in Figure 1 below, a map of the intense production regions in Greece and, in the Appendix A, a table of the production volumes is shown. Overall, nine open-ended interviews were conducted with C-level executives of the sustainable tomato sector either in specific (e.g., just producing) or multiple (e.g., producing and retailing) supply chain echelons. The expert selection was based on their long-term empirical participation in the food industry and their knowledge and understanding of the food network structure (i.e., purposive sampling method). Key features of our sample were—entities managing 6.000 sqm facilities, including 3.000 tons cold storage space with controlled atmosphere, 8 modern packaging lines, and 16 maintenance and cooling vehicles for product distribution; entities with 1.000 acres direct management and indirect cooperation, with more than 300 producers throughout Greece; entities that distribute products in over 1.000 end sale points throughout Greece, cooperate with the majority of the organic food stores like supermarket chains and which have more than 100 wholesale customers in Greece.

The research design pursued incorporates two methodological foundations—the approach of supply chain mapping that combines elements and techniques from value stream mapping [45] and value chain analysis [38], i.e., the case study approach [55]. The utilized research steps involved both secondary and primary research and were based on a four-stage abstraction process. Stage 1 created an understanding of the potential benefit, and eventual goal, of the mapping—mainly based on literature review and sectoral studies. In Stage 2 we developed the overall supply chain structure maps—based on the theoretical understanding gained from the previous stage. Stage 3 suggests the mapping of individual entities and interactions along the chain—verification, adjustment, and completion of the theoretical mapping based on open-end interviews. Finally, reporting and insights were generated in Stage 4.

## 3. Results and Discussion

**Figure 2:**
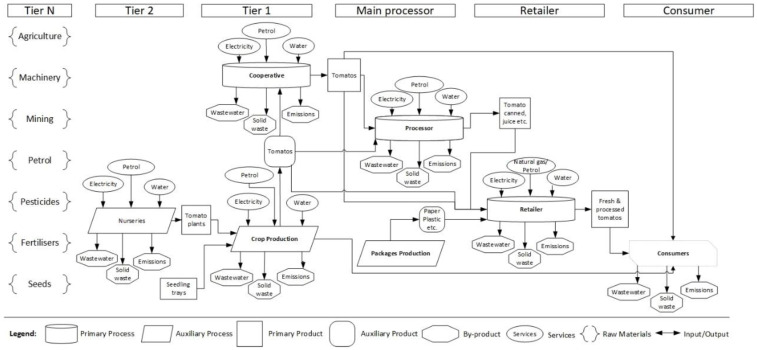
Tomato Supply Chain Structure Sustainability Input–Output.

**Figure 3:**
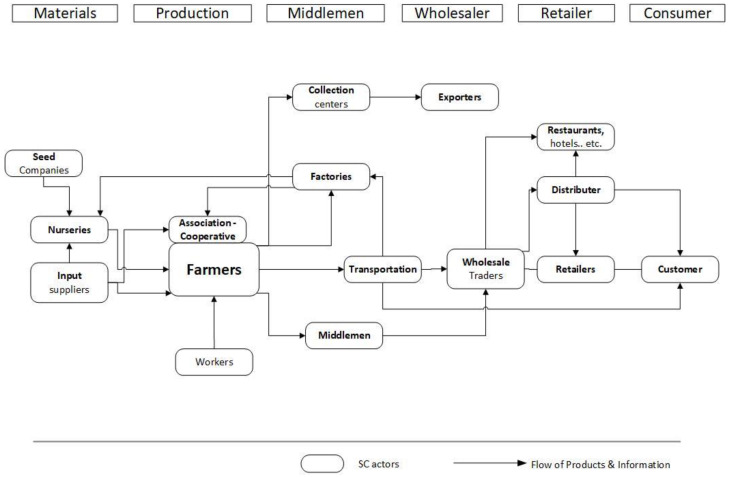
Tomato Value Chain.

**Figure 4:**
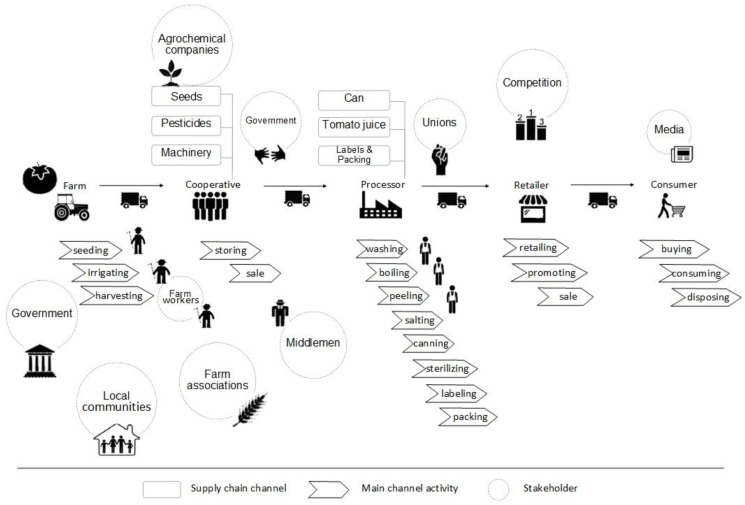
Tomato supply chain and stakeholders, adapted from Busse, Schleper, Weilenmann, and Wagner [23].

A better understanding of the Greek agri-food sector (stage 1) suggests a threefold mapping goal orientation. First, the recent Common Agricultural Policy (CAP) reform [57,58] provided encouragement and a clear need [8,28] for better adoption of sustainable practices along the supply chain, not only with regards to tomato but for the entire agri-food sector. Thus, the first mapping goal was a representation of the tomato supply chain structure focusing on sustainability (Figure 2) and mainly on its environmental pillar by diagramming the respective processes, services, different types of products, raw materials, and their input/output flows, for every supply chain tier (details in Section 3.1). Second, the development of organic farming in Greece in the past decade [51,53] and the growth of the tomato sector [2,3,4,5,52] dictate—as the selected method also implies by default—a value chain mapping (Figure 3 and Section 3.2), as a second mapping goal. Third, there is a fundamental role of the stakeholders in Greek agri-food sector [28] and in general food supply chains; for example, a proper flow of information among stakeholders results in detecting and preventing food integrity issues [59]. Consequently, the third mapping goal was to illustrate every process, unions, authorities, and stakeholders involved in the supply chain, including their interactions, critical points, the flow of products, and information (Figure 4 and Section 3.3).

### 3.1. Tomato Supply Chain Structure Sustainability Input–Output

The Tomato Supply Chain Structure Sustainability Input–Output map (Figure 2) is a visual representation of the processes, services, different types of products, raw materials, and their respective input/output flows for every supply chain tier. Specifically, all the raw materials involved in the tomato supply chain are in Tier N. This stage includes everything involved in the primary production, from the basic input like seeds, fertilizers, and pesticides to machinery, petrol, and all necessary materials, to have the entire production up and running. In Tier 1 and 2, there is a representation of the production phases, i.e., the nurseries to produce and deliver the plans, open-fields and greenhouse crop productions that grow and deliver fresh tomatoes, and farm cooperatives/associations dealing with postharvest treatment. The key actors involved in these stages were mainly farm workers and agrochemical companies. The next stages concern the processors and the retailers. Depending on the type of product, this could either be a processing facility for tomato juice, ketchup, canned tomatoes, etc., or just be packing for grocery shelves. Obviously, there are workers involved here as well, along with the middlemen, and there is also an important role for the government, several unions, and associations. The final stage is consumers, who can get different processed or fresh tomato products from retailers or directly from the producers. The map illustrates the primary and auxiliary processes, primary, auxiliary products and by-products, as well as the services and the relevant direction (as an input or output) among them.

Such mapping of the tomato supply chain structure could serve as a tool in identifying critical points concerning several sustainability issues. Primarily, this could be used for an initial assessment of resource efficiency issues by spotting the areas with excessive use of energy [60]. Similar to sustainability [61], resource intensity [62] and environmental [63] hotspot analysis—a conceptual understanding of the supply chain under investigation—is an essential first step. Likewise, in SAFA (Sustainability Assessment of Food and Agriculture Systems), methodology developed by FAO [64] a mapping analogous to the one developed in our study was among the required preparatory stages that was essential for the full assessment.

### 3.2. Tomato Value Chain

Τhe tomato value chain (Figure 3) describes the full range of stages and actions that are required to bring tomato/tomato-products from seed, through the different phases of production (involving processing, packaging, storing, transportation, and the input of various stakeholder services, i.e., middlemen, wholesalers, and retailers) to the final consumers. Starting with the materials stage, the main actor here is the nursery, whose key inputs are the seeds, and other supplies. Next in line is the production stage, with farmers, workers, and cooperatives/associations as the key players; while the product here is fresh tomatoes. This is followed by the middlemen stage which involves, besides the actual middlemen, the collection centers, factories, and transportation actors. From this stage onwards, the product flows involve either fresh or processed tomatoes. Next is the wholesaler stage, with the wholesalers and the exporters as actors. After this comes the retailer stage, with the key actor including different types of retailers (e.g., supermarkets and groceries), distributors, and outlets like restaurants, hotels, etc. The final stage is the consumer. However, here the objective was to illustrate the overall structure of the supply chain through the representation of all its actors and stakeholders, focusing on how they were interrelated via an exchange of products and information. In a similar way to value chain analysis [47], our mapping required the selection of a specific value stream; thus, the flow of products and information is the critical element in this map. The intention was to “cut a slice” through the complexity of the supply network to comprehend the key features of the current state [38].

The significance of this map for a research framework design was towards the identification of key points that could “block” a proper information flow linked to vital issues of the entire supply chain. As already highlighted in other sustainable supply chains, e.g., Greek organic citrus [25] and Dutch organic tomato [65], bottlenecks of information flows could have a significant impact on the entire supply chain performance. The essence of the mapping proposed in the current work was to produce a systemic map of the value chain via the systematic exploration of each activity concerning the consumer’s value [66]. This approach revealed the complex paths of products and information flow, allowing targeted interventions with a significant impact not only on the supply chain performance but also (and mainly) on the consumer.

### 3.3. Tomato Supply Chain and Stakeholders

The Tomato Supply Chain and Stakeholders map (Figure 4) refers to an illustration of every process and stakeholder involved in the supply chain, including their interactions, critical points, the flow of products, and information. Every supply chain channel, including a visual representation of their main channel activities, and a detailed association of every potential stakeholder is visually represented in this map. There is also a more detailed elaboration of the value chain map (see Section 3.2 and Figure 3) focusing on the stakeholders rather than on the information flow. As presented in Figure 4, the involvement of entities, such as government, local communities, and unions, is now more obvious. Specifically, the first two stages refer to the production of fresh tomatoes and the basic postharvest treatment; therefore, the key stakeholders were farmers, farm associations, workers, local communities, government, agrochemical companies, and middlemen. The main activities were seeding, irrigating, harvesting, storing, and sale. The next stage was processing, with the key stakeholders being the unions; while, the activities were washing, boiling, peeling, salting, canning, sterilizing, labeling, and packing. After this comes the retail stage with competition as a stakeholder, and retail, promoting, and sale as activities. The final stage, as always is the consumer; the key stakeholder here is media and main activities like buying, consuming, and disposing. Such mapping reveals the dynamics among the stakeholders and the different processes in every stage of the supply chain. Therefore, it could serve, for example, as a tool for a better assessment concerning the social and governmental pillars of sustainability.

The role of the supply chain stakeholders in the supply chain management is critical for many reasons; for instance, sharing of information [59], achieving traceability [67], and improving sustainability performance [28]. Therefore, designing future research that aims to impact interventions, requires a clear understanding of the stakeholders involved, as well as their interactions and interrelations. Aligned with the contribution of stakeholders in extending the supply chain visibility [23], the visualization in Figure 4 provides a mechanism to identify the critical areas in the Greek tomato supply chain.

Paraphrasing Aristotle’s quote “the beginning seems to be more than half of the whole” (Aristotle, Nicomachean Ethics 1098b) [68], we could argue that the three maps combined represented more than half of an impactful research framework; in the current scenario, this refers to the Greek tomato supply chain and eventually the entire agri-food sector. The remainder is an apposite interpretation that would set-up the research objectives and directions. Consequently, future research for the Greek tomato industry should begin with a comprehensive sustainability assessment, focusing on resource efficiency and the social (including, if not as a separate pillar, governmental) aspects. A careful look at the sustainability (Figure 2) and stakeholder (Figure 4) maps indicates numerous processes that require an extensive use of energy and water, involved in a complicated structure with multiple stakeholders and authorities. Contrary to a linear or myopic view only in specific elements, for example solely environmental performance, the proposed approach is more likely to eventually achieve an actual impact since, due to the wider perspective, it takes into consideration the influence of the stakeholders/authorities that could block or enhance practical interventions derived from the research output.

The clear goal to improve the sustainability performance is strongly linked to extended supply chain visibility and eventually to effective traceability of tomato/tomato-products throughout the supply chain [69]. The structure of supply chain, the processes involved, and the significant role of stakeholders as represented in the three maps (Figure 2, Figure 3 and Figure 4) underline the necessity for research to focus in these directions and vice-versa. The effective research for a traceability system relies on an extensive mapping that reveals the complexity and hidden interactions throughout the supply network. For instance, the value chain (Figure 3) and stakeholder (Figure 4) maps indicate the different routes in which a product could reach the final consumer and most importantly the reverse flow of information (e.g., consumer feedback, demand, and other insights) back into the supply chain. Thus, mapping reveals the pivotal role of the consumer in any research for and implementation of a successful traceability system. In agreement with previous studies [70,71] exploring consumer acceptance of a traceability system and their willingness to pay a price premium on the final products, this should be among the priorities of future research.

## 4. Conclusions

The three maps presented in the current work cover in full details the requirements for an impactful research framework design. The first map, tomato supply chain structure sustainability input–output (Section 3.1 and Figure 2), presents the different processes and provides additional information regarding their input–output, highlighting the critical points to be assessed in sustainability analysis. The second map, tomato value chain (Section 3.2. and Figure 3), shows the complicated flow of products and information, suggesting potential critical points, i.e., flow bottlenecks. Finally, the third map, tomato supply chain and stakeholders (Section 3.3 and Figure 4), focuses on the stakeholders and provides additional details regarding the overall understanding of the entire sustainable tomato supply chain. The synthesis of the output of the three maps provides a bigger picture and a deeper understanding of the Greek tomato supply chain. This wider end-to-end view of the supply chain is the added value of the current work.

Further analysis and discussion of the findings in the previous section, resulted in specific recommendations for the Greek tomato supply chain and consequently for the entire Greek agri-food sector. The underlined difference between the proposed focus on traceability and any other similar recommendations in the same direction (there are plenty in the last decade, see for example [67,69,70]), is the actual linkage with the social pillar of sustainability and most importantly with a consumer-centric supply chain orientation. Solely investigating the consumers’ willingness to pay (WTP) in relation to traceability does not provide as extensive insights as the advised suggestion in the current work; the end-to-end exploration of consumers understanding, including WTP, is linked to all stages of the supply chain, taking into consideration the social sustainability impact. The key difference is that the latter more thorough exploration is more likely to result in actual and applied output. Building on findings from previous studies on the importance of mapping [38,46,72] and its extensive use [17,36,48,73,74], our work highlights and exemplifies its application in research design.

The significance of the Greek tomato industry relies on its production volume (third among horticultural products), its processing potential (first in terms of processing volumes), and its export orientation [1,2,3]. Therefore, its contribution to the Greek agri-food sector and, as a result, to the Greek economy is noteworthy. The wider and deeper understanding of the entire supply chain is a crucial step in improving its sustainability performance [7]—in practice and not in theory—creating a significant competitive advantage, in this way [25]. Moreover, addressing sustainability and efficiency issues based on an extensive end-to-end mapping, such as the one proposed here, allows the researchers to work on every critical issue that could otherwise remain hidden. Thus, a significant improvement in issues like social sustainability could now be tackled more effectively; something that is a vital case for the Greek agri-food sector [8]. The managerial, practical, and policy-making implications that arise from the proposed approach go beyond the design of an impactful framework discussed above. Any meaningful intervention in an agri-food system—at different levels, e.g., strategy, performance, policy and so on—should start with a similar mapping.

## Appendix A

Based on data from the Hellenic Statistical Authority (ELSTAT) the table below presents the tomato production volumes in Greece.

**Table A1:** Tomato production volume. Source: ESTAT [56].

Production (Thousand Tons)	2011	2012	2013	2014	2012/11	2013/12	2014/13
Tomatoes (all)	1294.6	1234.3	1221.2	917.9	−4.7	−1.1	−24.8
Industrial	643.9	617	583.8	439.1	−4.2	−5.4	−24.8
Field	400.3	396.4	390.7	260.8	−1	−1.4	−33.2
Greenhouse	250.4	220.8	246.7	218	−11.8	11.7	−11.6
